# Modelling p-value distributions to improve theme-driven survival analysis of cancer transcriptome datasets

**DOI:** 10.1186/1471-2105-11-19

**Published:** 2010-01-11

**Authors:** Esteban Czwan, Benedikt Brors, David Kipling

**Affiliations:** 1 School of Medicine, Cardiff University, Heath Park, Cardiff CF14 4XN, UK; 2 Theoretical Bioinformatics, German Cancer Research Center, Im Neuenheimer Feld 280, 69120 Heidelberg, Germany

## Abstract

**Background:**

Theme-driven cancer survival studies address whether the expression signature of genes related to a biological process can predict patient survival time. Although this should ideally be achieved by testing two separate null hypotheses, current methods treat both hypotheses as one. The first test should assess whether a geneset, independent of its composition, is associated with prognosis (frequently done with a survival test). The second test then verifies whether the theme of the geneset is relevant (usually done with an empirical test that compares the geneset of interest with random genesets). Current methods do not test this second null hypothesis because it has been assumed that the distribution of p-values for random genesets (when tested against the first null hypothesis) is uniform. Here we demonstrate that such an assumption is generally incorrect and consequently, such methods may erroneously associate the biology of a particular geneset with cancer prognosis.

**Results:**

To assess the impact of non-uniform distributions for random genesets in such studies, an automated theme-driven method was developed. This method empirically approximates the p-value distribution of sets of unrelated genes based on a permutation approach, and tests whether predefined sets of biologically-related genes are associated with survival. The results from a comparison with a published theme-driven approach revealed non-uniform distributions, suggesting a significant problem exists with false positive rates in the original study. When applied to two public cancer datasets our technique revealed novel ontological categories with prognostic power, including significant correlations between "fatty acid metabolism" with overall survival in breast cancer, as well as "receptor mediated endocytosis", "brain development", "apical plasma membrane" and "MAPK signaling pathway" with overall survival in lung cancer.

**Conclusions:**

Current methods of theme-driven survival studies assume uniformity of p-values for random genesets, which can lead to false conclusions. Our approach provides a method to correct for this pitfall, and provides a novel route to identifying higher-level biological themes and pathways with prognostic power in clinical microarray datasets.

## Background

In clinical oncology, the discovery of knowledge from gene expression microarray experiments is often based on what has been described a "top-down" or hypothesis-free approach, where a prognostic model is derived from global tumour gene expression data in a way that does not require *a priori *knowledge of biological function [1]. A subsequent goal is to extract one or more higher-level biological "themes" from this primary model. In practice the task of inferring themes regarding biological function from a prognostic geneset, which may consist of a relatively small number of genes representing very distinct biological processes, has proved to be a major analytical bottleneck.

A contrasting approach is a "bottom-up" or hypothesis-driven method where sets of biologically-related genes (e.g. all transcripts coding for proteins of a particular signalling pathway) are defined first, and then these sets of genes are analysed to determine if any set acts as a prognostic indicator. Since the biological functions of the genesets are already known, sets with prognostic power can then play a more informative role in clinical decision-making [1]. We will refer to this particular approach as "theme-driven", where the biological function of a geneset becomes a theme, so as to distinguish it from other geneset-based methods in which genes do not share common functionality. Although useful in principle, a theme-driven approach in clinical oncology has not been widely used until recently, mainly due to the difficulty of defining sets of genes of common cellular function. However, the emergence of function-oriented classifications of genes, such as those developed by the Biocarta [2], Gene Ontology (GO) [3], Gene Map Annotator and Pathway Profiler (GenMAPP) [4], and Kyoto Encyclopedia of Genes and Genomes (KEGG) [5] consortia, in addition to those commercially available (e.g. Ingenuity [6], GeneGO [7]), has facilitated the comprehensive analysis of collections of biologically-related genes.

Common methods of theme-driven cancer survival analysis can involve clustering to segregate cancer samples into two groups according to the gene expression levels of the geneset of interest, and a subsequent survival test to determine whether a difference in prognosis exists between both groups [8,9]. It is important to clarify what it is being tested in such studies. This type of experiments has two inherent null hypotheses. Goeman and Bühlmann [10] have discussed these two very different hypotheses, which result from analysing genesets in terms of subject-sampling and gene-sampling models. Although discussed in a different context, the implications are relevant to any geneset analysis of gene expression microarray data. According to the authors, in a subject-sampling model the subjects (i.e. tissue samples or patients) are treated as the sampling units, and it is natural to formulate a self-contained null hypothesis. In the authors' example, this null hypothesis states that a geneset is not differentially expressed. A significant p-value against this self-contained null hypothesis would mean that the same association found for the subjects analysed would also be found for a new set of subjects with high certainty. Yet, this p-value does not make any statement about how the geneset relates to others. On the other hand, in a gene-sampling model the genes become the sampling units, and it is straightforward to formulate a competitive null hypothesis. This hypothesis states that a particular geneset is differentially expressed as often as other genesets. In this case, a significant p-value would mean that the geneset would still be differentially expressed for the same subjects when new sets of genes are included on a new microarray. However, this p-value does not imply that the same association would be found for a new set of subjects. Throughout this paper, the expressions p-value_1 _and p-value_2 _will refer to p-values obtained from the first (self-contained) and the second (competitive) hypothesis tests, respectively. The expression p-value will be used in general terms.

In theme-driven survival studies, the two null hypotheses (with an exception mentioned below) must be tested. The first null hypothesis, which is self-contained and based on subject-sampling, claims that a particular geneset, independent of its composition (i.e. made up of either biologically-related or random genes), has no predictive power of survival time. A survival test, such as a log-rank test, is the usual choice for testing this hypothesis. A significant p-value_1 _for a geneset would give one confidence that the association between the geneset and survival would also be found for new patients, but it does not demonstrate that the theme of the geneset is actually relevant. This is addressed with a competitive null hypothesis based on gene-sampling. This second null hypothesis states that a set of biologically-related genes is, at most, as correlated with survival as are random sets of unrelated genes. This hypothesis can be tested by an empirical test that compares the correlation to survival of a given geneset with that of random sets of unrelated genes. A significant p-value_2 _against this hypothesis would mean that the theme of the geneset is associated with survival time, but only for the patients analysed in the dataset. Therefore, both null hypotheses must be rejected in order to claim predictive power of a theme.

If the distribution of p-values_1 _for random sets of unrelated genes is uniform, which in turn implies that most sets of unrelated genes are non-informative with respect to survival, testing for the second null hypothesis becomes redundant. To illustrate this point with an example, assume that a biologically-related geneset *g *has prognostic power as assessed by rejecting the first null hypothesis at a p-value_1 _*p *= 0.004 below a significance threshold *α*. If this geneset is systematically compared to *n *= 1000 non-informative (random) genesets (i.e. testing the second null hypothesis), it will outperform them approximately (1 - *p*) × *n *= 996 times (assuming a uniform distribution of p-values_1 _for non-informative genesets). It is expected that 4 random genesets will be at least as significant as geneset *g*, which means that the empirical p-value_2 _for *g *will also be equal to 4/1000, that is, 0.004 (see Methods). Since p-value_1 _and p-value_2 _are equal, it would be enough to only test the first null hypothesis. However, it has been reported that the distribution of p-values is often not uniform in gene expression experiments [11], which questions the adequacy of current theme-driven survival methods that assume uniformity. In our study, we are therefore interested in the distribution of p-values_1 _for random sets of unrelated genes, since by incorrectly assuming a uniform distribution of these p-values_1_, a researcher may falsely conclude that the theme of a geneset has prognostic power.

One situation that may lead to false positive conclusions in theme-driven survival analysis is when there are widespread changes in gene expression that lead to the patient samples clustering in a prognostically informative manner. Take as an extreme example a dataset consisting of two tumour types: benign early lesions and aggressive metastatic disease. Assume that the degree of transcriptome changes is so great that the data for any randomly selected group of genes clusters in such a way as to separate the patients into benign and aggressive categories. In such a scenario any biologically-based geneset (say all the genes within a particular GO category) might likewise cause an informative clustering of the data, and thus have prognostic power. The erroneous conclusion would be that this particular GO geneset has prognostic power greater than a random geneset, that is, the biological process (i.e. the theme of this geneset) is associated with prognosis. Formally speaking, the error has been caused by assumptions regarding the distribution of the p-values_1 _for random sets of unrelated genes.

Although this hypothetical experiment is somewhat extreme, it illustrates the caution that must be taken when interpreting the p-values from simple applications of theme-driven survival techniques. The key to a more rigorous approach is to model the distribution of p-values_1 _for random genesets, so as to then also provide accurate empirical p-values_2 _for the biologically-related genesets. Although some hypothesis-free studies [12,13] and some geneset-based experiments focusing on differential expression patterns [14] have implicitly dealt with the possible non-uniform distribution of p-values_1 _for random genesets, theme-driven cancer survival studies have not, to date, acknowledged this problem.

Therefore, in this study we set out to investigate the distribution of p-values_1 _for random sets of unrelated genes and its effects on theme-driven cancer survival methods. Using two publicly available datasets we demonstrate that such distribution is indeed non-uniform. We then describe a method that empirically approximates this distribution using a permutation approach based on 100,000 random sets of unrelated genes. Finally, we apply this methodology to these two datasets and compare our results with those of a previous theme-driven study [8]. This study has reported a significant association of a gene expression signature of fibroblasts in response to serum (core serum response, CSR, signature as called by the authors) with patient survival for many cancer datasets, including the breast and lung cancer sets used in our study. Our data question the validity of some of those associations, since the method employed in such study assumes that p-values_1 _for random genesets are uniformly distributed. However, our data reveal that such assumption is frequently incorrect, which may result in erroneous conclusions, and we provide an empirical methodology to address this problem.

## Results

**Figure 1:**
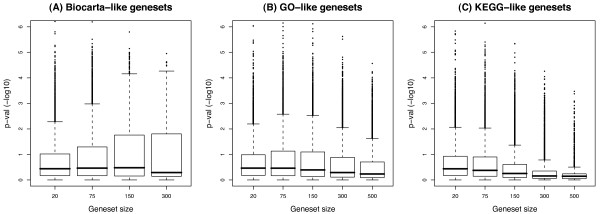
**Association between geneset size and significance for breast cancer OS**. For each type of geneset (i.e. Biocarta, GO, and KEGG), sets of random genes of sizes 20, 75, 150, 300, and 500 (the latter omitted for Biocarta because the pool of genes was too small) consisting of 10,000 genesets for each size were generated. For each geneset, hierarchical clustering was performed to segregate samples into two groups, a subsequent log-rank test was performed to assess a difference in prognosis between both groups, and the p-value_1 _was recorded. The negative base 10 logarithms of the p-values_1 _are plotted against geneset size. Biocarta-like genesets appear to be more significant around length = 150 (A); GO-like genesets do not show a clear correlation of significance to geneset size (B); KEGG-like genesets seem to be more significant as size becomes smaller (C).

Each original geneset was tested against the first and the second null hypotheses. For each geneset, cancer patients from a breast cancer gene expression study and a lung cancer study were split in two clusters based on the expression values of the genes in the geneset, and a p-value *p*_1 _was calculated by comparing survival times for patients in each cluster with a log-rank test (first hypothesis test). By taking 100,000 randomly permuted sets of unrelated genes with the same geneset size distribution as Biocarta, GO, KEGG, and CSR genesets, we calculated empirical distributions of p-values_1 _for random genesets. Since an association is present between geneset size and significance (Figure 1, Additional Files 1, 2), the sizes of the randomly generated genesets were matched to those of the original genesets so as to include the same proportion of geneset sizes in each empirical distribution. For each original geneset, *p*_1 _was compared with the random p-values_1 _of the appropriate distribution and an empirical p-value *p*_2 _was calculated (second hypothesis test). A false discovery rate (FDR) method was implemented to control for multiple testing for each hypothesis test, namely *FDR*_1 _and *FDR*_2 _for the first and second hypothesis tests, respectively.

### Non-uniform distributions for random genesets

**Figure 2:**
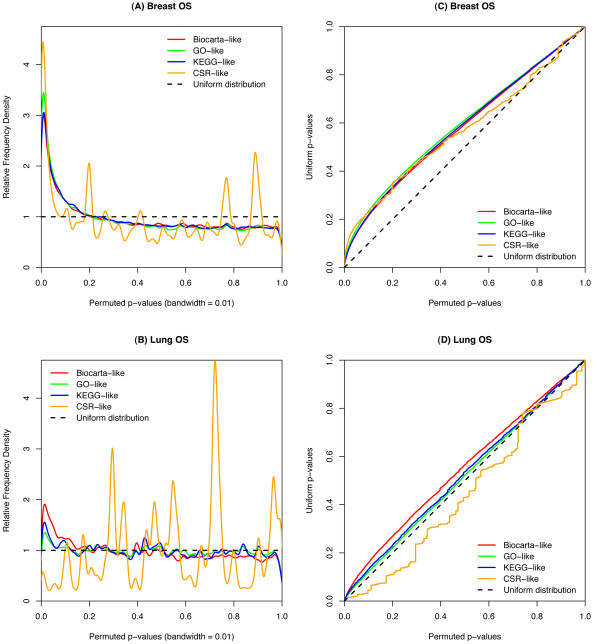
**Empirical p-value**_1_**distributions of random genesets for breast cancer OS and lung cancer OS**. Relative frequency density distributions are shown for breast cancer OS (A) and for lung OS (B). For each survival estimate (i.e. breast cancer OS, and lung cancer OS), the relative frequency density estimate with a bandwidth equal to 0.01 is plotted for each empirical distribution (i.e. Biocarta-like, GO-like, KEGG-like, and CSR-like). A uniformly distributed empirical distribution would result in p-values_1 _at the same frequency across the entire range 0 < p < 1 (dashed line in (A) and (B)). Ordered plots of empirical p-value_1 _distributions versus the uniform distribution are shown for breast cancer OS (C) and for lung cancer OS (D). The permuted p-values used to model each distribution (i.e. Biocarta-like, GO-like, KEGG-like, and CSR-like) are plotted against random p-values_1 _from a uniform distribution. An x = y line would be expected if the empirical distributions were uniformly distributed.

As shown in Figure 2, the four empirical distributions of random genesets (i.e. Biocarta-like, GO-like, KEGG-like, and CSR-like) for both breast and lung overall survival (OS), as well as for breast relapse-free survival (RFS, Additional File 3), deviate considerably from the expected uniform distribution. Figure 2A and Figure 2B show the density distributions of p-values_1 _for breast OS and lung OS, respectively (Additional File 1A shows the density distributions for breast RFS). Additional File 4 shows the same density distributions for breast OS and lung OS but on negative logarithmic scale. It can be appreciated that the peaks of most distributions occur around 2, which represents a p-value_1 _of 0.01 (a commonly used significance threshold). A uniform distribution would mean that p-values_1 _should be seen at the same frequency across the entire range 0 <*p *< 1, hence a straight horizontal line (i.e. uniform distribution) is expected. That this is not seen suggests that p-values_1 _for random genesets are not uniformly distributed in these two examples. Instead, there is an excess number of random genesets scored as apparently significant (for example, with *p *< 0.01). In addition Figure 2C and Figure 2D also show the non-uniformity of p-values_1 _for random genesets for breast OS and lung OS, respectively (Additional File 3B shows these results for breast RFS). In these plots an x = y line would be expected if the empirical distributions were uniformly distributed, which is clearly not seen.

### First hypothesis and second hypothesis tests

Table 1 shows a summary of results for the original genesets and their putative association with cancer prognosis. First hypothesis tests returned 66 (of the 1,082 genesets tested) as being significant for breast cancer OS, 58 for breast cancer RFS, and 32 (of the 1,426 genesets tested) for lung cancer OS with p-values *p*_1 _< 0.01. By way of illustration, 1,082 random genesets typically returned 61 and 55, for breast OS and RFS respectively, as being significant at the same threshold (despite only less than 11 being expected by chance at this p-value threshold). When the original genesets were tested against the second null hypothesis, only 7 and 13 genesets were significant for breast cancer OS and RFS, respectively (*p*_2 _< 0.01). On the other hand, 18 genesets were significant (*p*_2 _< 0.01) for lung cancer OS.

**Table 1:** Summary of results.

Survival Estimate	Number of genesets (*p*_1_< 0.01)	Number of genesets (*p*_1_< 0.01; *FDR*_1_< 0.30)	Number of genesets (*p*_2_< 0.01)	Number of genesets (*p*_2_< 0.01;*FDR*_2_< 0.30)
Breast OS	66	66	7	1
Breast RFS	58	58	13	0
Lung OS	32	20	18	4

For each survival estimate (i.e. breast OS, breast RFS, and lung OS), the numbers of significant genesets against the first null hypothesis (*p*_1 _< 0.01) and against the second null hypothesis (*p*_2 _< 0.01), as well as the numbers of genesets with *FDR *values below the significant threshold (*FDR *< 0.30) for those significant genesets are shown.

### Novel significant genesets

Table 2 describes the genesets that were significant for both hypothesis tests and remained statistically significant after correction for multiple testing (*p*_1 _< 0.01; *FDR*_1 _< 0.30; *p*_2 _< 0.01; *FDR*_2 _< 0.30). Additional Files 5, 6, 7, 8, and 9 provide with the identities and descriptions of the genes of each significant geneset.

**Table 2:** Significant genesets and CSR comparison.

Geneset Name	Survival Estimate	Chang *et al*.(p-value)	*p*_1_	*FDR*_1_	*p*_2_	*FDR*_2_
GO "fatty acid metabolism"	Breast OS	-	**0.0000005**	0.006	**0.00014**	0.30
CSR	Breast OS	0.0410	0.0321000	0.280	0.14929	0.99
CSR	Breast RFS	0.0130	0.0144000	0.210	0.09614	0.99
GO "receptor mediated endocytosis"	Lung OS	-	**0.0000020**	0.003	**0.00003**	0.02
GO "brain development"	Lung OS	-	**0.0000020**	0.003	**0.00003**	0.02
GO "apical plasma membrane"	Lung OS	-	**0.0001020**	0.040	**0.00078**	0.29
KEGG "MAPK signaling pathway"	Lung OS	-	**0.0001020**	0.040	**0.00080**	0.29
CSR	Lung OS	0.0014	**0.0026000**	0.230	**0.00325**	0.53

For each survival estimate, a description of the significant biologically-related genesets (*p*_1 _< 0.01; *FDR*_1 _< 0.30; *p*_2 _< 0.01; *FDR*_2 _< 0.30) as well as a comparison of the p-values reported by Chang *et al*. and our log-rank (*p*_1_) and empirical (*p*_2_) p-values for the CSR gene expression signature are shown.

### Revision of Chang et al. results

We then compared our methodology with that of Chang *et al*., who reported a significant association (*p *< 0.05) between the CSR signature and survival (Table 2) by testing only the first null hypothesis. As might be expected, our p-values *p*_1 _differ slightly from those obtained by Chang *et al*., but in each case also appear significant (*p*_1 _< 0.05). This confirms that the CSR signature is associated with survival time. However, these results cannot be used to support any claim that the biological process of the signature is also correlated with cancer prognosis. In fact, when testing for the second null hypothesis, two of the three estimates are not significant. Although the response of fibroblasts to serum appears to be highly correlated to lung cancer OS (*p*_2 _< 0.00325), the empirical p-values_2 _for CSR with regards to breast cancer OS (*p*_2 _< 0.14929) and RFS (*p*_2 _< 0.09614) do not reach statistical significance, casting severe doubts on the association of the response of fibroblasts to serum with breast cancer OS and RFS. This example illustrates how the non-uniform distribution of p-values_1 _for random genesets can lead to false positives when using a simple statistical approach to theme-driven survival studies.

## Discussion

Unlike hypothesis-free studies in survival analysis, theme-driven experiments aim to test whether a set of biologically-related genes is associated with survival. In other words, researchers seek an association between the theme of the geneset and cancer prognosis. This implies that such predefined genesets must be significant predictors as determined by a survival test, which constitutes the first null hypothesis, and also must perform "better" than most sets of unrelated genes, which constitutes the second null hypothesis. If the distribution of p-values_1 _for random genesets is uniform, which implies that most genes in gene expression datasets are uninformative with respect to survival time, then the second null hypothesis need not to be tested. However this is rarely the case; p-values_1 _are usually non-uniformly distributed.. Therefore, any attempt to determine significance of the theme of a geneset based on the first hypothesis test is rendered meaningless [11]. Our results illustrate this (Figure 2), and raise the issue that simple methods in theme-driven survival studies that assume uniformity of p-values_1 _for random genesets may yield false conclusions. Even when an empirical distribution is used to calculate empirical p-values_2 _for the original themes of interest, it must be noted that such p-values_2 _do not claim anything about the predictive power of the themes for new sets of patients. Theoretically, this claim has to be assessed by a prior (or posterior) independent hypothesis test that verifies an association between a geneset and survival time. In our study, such hypothesis testing has been done beforehand when a p-value_1 _was calculated for each geneset with a log-rank test. It is arguable that such a simple test can have predictive power for new patients, but it is a common procedure in survival analysis and a thorough examination of this topic is outside the scope of this paper.

In the analysed breast cancer dataset, many genesets were correlated to cancer prognosis when considering p-values_1 _from the first hypothesis test, both for OS and for RFS. After testing the second null hypothesis, most of these genesets did not reach statistical significance. This is consistent with the skew towards low p-values_1 _of the empirical distributions for random genesets (Figure 2A). Conversely, the lung cancer dataset identified several genesets that were significant for both the first and second hypothesis tests. This is in turn consistent with the lesser skew towards low p-values_1 _(Figure 2B).

Chang *et al*. have reported a correlation of their core serum response (CSR) gene expression signature to survival time in many cancer datasets, including the same breast and lung cancer sets used in our study. As with other theme-driven approaches, these authors did not consider the impact of non-uniformly distributed p-values_1 _for random genesets, so they tested only the first null hypothesis. Our findings suggest that this may have biased their results, leading to false correlations of the CSR signature to cancer survival. While it remains possible that this geneset is a significant predictor of survival in new patients for breast cancer (rejection of the first null hypothesis), it cannot be claimed that the biology of that geneset is relevant, given that many random genesets achieve at least the same predictive power (acceptance of the second null hypothesis). Our empirical approach improves upon their methodology and provides a technique to avoid this pitfall.

Although we have illustrated this issue using some commonly available ontologies, it is important to note that current biological databases still offer a somewhat limited perspective with respect to the annotation of gene function. Although biological ontologies (e.g. KEGG, Biocarta, GO) have successfully grouped genes in terms of biological function, such lists do not account for relationships and dependencies among genes. Moreover, different databases have overlapping annotations, hence analysing genesets from various ontologies may result in redundant tests due to duplication or multiplication of genesets. In principle our methodology can be applied equally well to other geneset collections, including proprietary sets and those available through commercial providers.

In this study we used an FDR method to control for multiple testing, which assumes that the test statistics are only weakly correlated [15]. This is conservative, because it does not account for the strong dependencies and correlations in our genesets. Filtering by use of a non-stringent p-value (or FDR value) has been suggested, since screening studies with small sample sizes will have inherently unstable feature rankings in any statistical test; thus, reproducibility of gene expression microarray experiments will be increased by more permissive thresholds [16], which is particularly important for theme-driven cancer survival studies. Many genesets that were significant for both hypothesis tests and also after correction for multiple testing are biologically plausible in the light of long-established biological knowledge and recently published studies. Our results show a correlation of the GO term "fatty acid metabolism" to breast cancer overall survival. This mirrors a recent experimental finding that reports an association between the failure to respond to tamoxifen treatment and the expression patterns of genes involved in cholesterol and fatty acid metabolism for estrogen receptor positive (ER+) breast tumours [17]. Our data also revealed an association between the GO term "receptor mediated endocytosis" and overall survival time in lung cancer. Again, the importance of proteins involved in receptor mediated endocytosis in lung cancer development, progression, and metastasis has been discussed in the literature [18]. Our results show additionally a relationship between the KEGG pathway "MAPK signaling" and overall survival in lung cancer. This is a confirmation of the well-known oncogenic properties of the MAPK signaling pathway, whose role in cancer development and progression have been extensively addressed in the literature for lung and many other cancers [19-21]. Lastly, our data suggests a correlation of the GO term "apical plasma membrane" with lung cancer overall survival. Researchers have reported that lipid trafficking to the apical plasma membrane of pulmonary epithelial cells is thought to be a protective stress response [22]. It can be speculated that lung cancer cells may develop by deregulation of this mechanism.

## Conclusions

Our results show that p-values_1 _for random genesets in theme-driven survival studies are frequently not uniformly distributed. This impairs common-practice methods, and results in false positive associations between the theme of a geneset and survival. Our proposed empirical approach correctly avoids such pitfalls, and the plausible biological observations obtained suggest that a theme-driven method that correctly tests the two inherent null hypotheses can contribute to further understanding of the biology of cancer (and other clinical survival datasets), and help bridge the gap between gene expression predictors and biological knowledge.

## Methods

### Description of datasets

The study utilizes two publicly available cancer datasets. The first is a subset of the breast cancer dataset collected at The Norwegian Radium Hospital, Oslo [23]. The original dataset contained 85 tissue samples representing 84 patients, including 78 breast carcinomas, 3 fibroadenomas, and 4 normal breast tissue samples. Given that a common interest in theme-driven experiments is to distinguish subtypes of tumours, only data from a 51-sample subset of locally advanced breast carcinomas were used in the current study. The gene expression data of these 51 samples were downloaded from the Stanford Microarray Database (SMD) [24]. The clinical data were downloaded from the web supplement related to this dataset.

We also obtained a lung cancer dataset collected at the Howard Hughes Medical Institute [25]. The original dataset consisted of 67 human lung tumours and 6 normal lung tissue samples representing 56 individuals. All tumour samples were used in this study. The gene expression data of the 67 samples were downloaded from SMD. The clinical data were downloaded from the web supplement related to this dataset.

### Dataset preprocessing

The gene expression data for each tissue sample of the breast dataset were retrieved in separate Excel spreadsheets (one spreadsheet per sample) from SMD. All blank probes (i.e. the empty spots on the array) as well as all customised non-mappable probes were discarded (since genes in each geneset were represented by Entrez Gene accession numbers, it would have been difficult to include customised probes since no mapping tool converts those to Entrez Gene accession numbers). As a result, only those probes that were mappable IMAGE clones remained in the filtered dataset. Some clones referred to mitochondrial genes and were also discarded. Only adequately measured probes were used in this experiment, defined as a probe with fluorescent hybridization signals at least 1.5-fold greater than the local background signal in the reference channel. For each adequately measured probe, the normalised log ratio to the base 2 was extracted, whereas for inadequate probes a missing value (NA) was assigned. Since the dataset was derived from four distinct batches of cDNA microarrays, some probes only had values for a subset of samples where such probes were unique to the array type of those samples. In the cases where arrays contained replicated probes (i.e. the same IMAGE clone identifiers) a single average log ratio was used. Only those probes for which technically adequate measurements were obtained from at least 60% of the samples were used [8]. To correct for any systematic bias resulting from different amounts of cDNA in the reference channel, the probes of the gene expression subset were median centred (i.e. the gene-wise median expression value of a probe was subtracted from each log-ratio in the probe).

A similar procedure was carried out for the lung cancer dataset, with the only difference being the number of microarray types from which the dataset was derived and the number of probes in the arrays. The lung dataset came from six different batches of cDNA microarrays but, as opposed to the breast dataset, all six microarray types had approximately the same set of probes. For this reason it was possible to use a stricter criterion for filtering probes, with 80% of adequate measurements being required for a probe to be used [8].

Both the breast and lung cancer datasets were accompanied by extensive clinical data. Information relevant to this study included the overall survival (OS) time and its status (alive versus dead) for both datasets, with relapse free survival (RFS) time and its status (no relapse versus relapse) also being available for the breast cancer dataset. The OS status of the breast dataset was simplified from four different values (0 = no evidence of disease, NED; 1 = alive with disease, AWD; 2 = dead of disease, DOD; 3 = dead of other causes, DOC) to two (0 = NED, AWD, DOC; 1 = DOD), since our study aim was to focus on patient survival.

### Description of genesets

Predefined sets of genes were constructed from three distinct functional annotation groups: Biocarta pathways, KEGG pathways, and GO terms. The genesets were obtained from the human gene annotation file hosted at the Cancer Genome Anatomy Project (CGAP) web site [26]. For each functional group, all pathways or terms were retrieved along with all genes (Entrez Gene identifiers) that belonged to them. As a result, 179 genesets from KEGG, 314 from Biocarta, and 6,032 from GO were generated. One other set included was the original Chang *et al*. gene expression signature of fibroblasts in response to serum (CSR as defined by the authors), which was the only geneset made up of IMAGE clone identifiers.

Genesets that contained small (< 5) or very substantial (> 1,000) numbers of genes were excluded, as they are likely to be of limited biological utility. Entrez Gene accession numbers were converted to IMAGE clone identifiers using the web tool Clone/Gene ID Converter [27]. A geneset was also discarded if its genes were mapped to less than 5 or more than 2,500 probes. Additionally genesets that resulted in very uneven clusters were discarded (see next section), as well as a small number of genesets whose corresponding data was difficult to cluster because of excessive numbers of missing values. Overall, 1,082 genesets were tested in the breast cancer dataset, and 1,426 in the lung cancer set.

### Patient classification and initial statistical analyses

Patient classification and statistical analyses were performed with R (version 2.7) [28]. For each geneset and each cancer dataset, the gene expression values of the geneset probes (i.e. mapped genes) that were present in the cancer dataset were retrieved. The samples of this subset of gene expression data were clustered by average linkage clustering using a correlation-based distance matrix *d_xy _*defined by:

where *ρ*_xy _is the Pearson's correlation coefficient of *x *and *y*. In some cases, clustering could not be performed because missing values in the gene expression data would have generated missing values in the distance matrix. Since all pairwise distances are needed to cluster the data, any geneset that produced a distance matrix with missing values was discarded. After clustering, samples were segregated into two groups based on the first bifurcation of the hierarchical sample dendrogram, and only cancer samples with available clinical data were used to perform survival analysis.

Correlation of the clustered groups of patients to survival time was assessed using a log-rank test (first hypothesis test). Although many researchers have chosen to use a log-rank test in their survival studies, it has been shown that this test breaks down with small sample sizes, such as when at least one cluster or group of patients consists of very few samples [29]. To avoid extremely uneven clusters we discarded genesets where less than 10% of the samples were in the smaller cluster. For each survival estimate (i.e. breast OS, breast RFS, and lung OS), a p-value *p*_1 _was calculated.

### Empirical distributions of p-values

A distribution of p-values_1 _for random genesets was approximated for each type of geneset (i.e. Biocarta, GO, and KEGG) and for each survival estimate (i.e. breast OS, breast RFS, and lung OS) using 100,000 random sets of unrelated genes, and their prognostic power was assessed using the above methods (i.e. hierarchical clustering and a log-rank test). The number of random genesets was chosen according to a stability analysis which showed that 100,000 permutations were enough for the purposes of our study (Additional Files 10, 11, 12). The permutation results were used to generate "Biocarta-like", "GO-like", and "KEGG-like" empirical distributions of p-values_1 _for both breast and lung cancer datasets. A distinction was made for the three different types of genesets because the "universe" of genes (i.e. the genes that have an annotation) is not the same. For example, the universe of GO genes contains approximately 14,000 genes, whereas the universe of Biocarta genes only has around 3,000 genes. Selecting genes from a wider, or more restricted, universe than the real one may result in biased estimates [30]. Additionally, since an association is present between geneset size and significance (Figure 1, Additional Files 1, 2), the sizes of the randomly generated genesets were matched to those of the original genesets so as to include the same proportion of geneset sizes in each distribution.

Association between the theme of a geneset and survival time was assessed with an empirical test based on the previously modelled distributions (second hypothesis test). An empirical p-value *p*_2 _was calculated for each survival estimate for the original 1,082 and 1,426 genesets analysed in breast and lung cancer, respectively, which was defined by:

where  is the number of random genesets from the appropriate empirical distribution which yielded p-values less than or equal to the p-value *p*_1 _obtained from the log-rank test for the geneset of interest at the first hypothesis test, and  is the total number of random genesets. To control for the multiple testing of genesets in each hypothesis test we used a false discovery rate (FDR) method, defined as the proportion of expected false positive findings over the total number of alternative hypotheses accepted at the specified significance level [31]. *FDR*_1 _and *FDR*_2 _values were calculated for p-values of the first and second hypothesis tests, respectively.

In order to compare our CSR signature results to those of Chang *et al*., three CSR-like empirical distributions of p-values_1 _for random genesets (breast cancer OS, breast cancer RFS, and lung cancer OS), were constructed from 100,000 random sets of unrelated genes. For each distribution, the randomly generated genesets were chosen to match the size of the CSR signature. Furthermore, the universe of genes was designed so as to only include genes that could have been selected in the original experiment, based on the procedures described by Chang *et al*.

## Authors' contributions

DK conceived of and coordinated the study. EC, BB, and DK designed the study. EC carried out the data analyses and drafted the manuscript. All authors contributed to the preparation of the manuscript, and discussions on the topic of this paper. All authors read and approved the final manuscript.

## Supplementary Material

Additional file 1 **Association between geneset size and significance for breast cancer RFS**. For each type of geneset (i.e. Biocarta, GO, and KEGG), sets of random genes of sizes 20, 75, 150, 300, and 500 (the latter omitted for Biocarta because the pool of genes was too small) consisting of 10,000 genesets for each size were generated. For each geneset, hierarchical clustering was performed to segregate samples into two groups, a subsequent log-rank test was performed to assess a difference in prognosis between both groups, and the p-value_1 _was recorded. The negative base 10 logarithms of the p-values_1 _are plotted against geneset size. Biocarta-like genesets appear to be more significant around length = 150 (A); GO-like genesets do not show a clear correlation of significance to geneset size (B); KEGG-like genesets seem to be more significant as size becomes smaller (C). Click here for file

Additional file 2 **Association between geneset size and significance for lung cancer OS**. For each type of geneset (i.e. Biocarta, GO, and KEGG), sets of random genes of sizes 20, 75, 150, 300, and 500 (the latter omitted for Biocarta because the pool of genes was too small) consisting of 10,000 genesets for each size were generated. For each geneset, hierarchical clustering was performed to segregate samples into two groups, a subsequent log-rank test was performed to assess a difference in prognosis between both groups, and the p-value_1 _was recorded. The negative base 10 logarithms of the p-values_1 _are plotted against geneset size. Biocarta-like genesets seem to be more significant as size becomes smaller (A); GO-like genesets seem to be more significant as size becomes smaller (B); KEGG-like genesets seem to be more significant as size becomes smaller (C). Click here for file

Additional file 3 **Empirical p-value**_1_**distributions of random genesets for breast cancer RFS**. Relative frequency density distributions are shown for breast cancer RFS (A). The relative frequency density estimate with a bandwidth equal to 0.01 is plotted for each empirical distribution (i.e. Biocarta-like, GO-like, KEGG-like, and CSR-like). A uniformly distributed empirical distribution would result in p-values_1 _at the same frequency across the entire range 0 < p < 1, (dashed line in (A)). An ordered plot of empirical p-value_1 _distributions versus the uniform distribution is shown for breast cancer RFS (B). The permuted p-values_1 _used to model each distribution (i.e. Biocarta-like, GO-like, KEGG-like, and CSR-like) are plotted against random p-values from a uniform distribution. An x = y line would be expected if the empirical distributions were uniformly distributed. Click here for file

Additional file 4 **Empirical p-value density distributions of random genesets for breast cancer OS and lung cancer OS (negative logarithmic scale)**. Relative frequency density distributions are shown for breast cancer OS (A) and for lung OS (B). For each survival estimate (i.e. breast cancer OS, and lung cancer OS), the relative frequency density estimate with a bandwidth equal to 0.01 is plotted for each empirical distribution (i.e. Biocarta-like, GO-like, KEGG-like, and CSR-like) on a negative logarithmic to the base 10 scale. A uniformly distributed empirical distribution would result in p-values_1 _at the same frequency across the entire range -log(0) > -log(p) > -log(1) (dashed line in (A) and (B)). Click here for file

Additional file 5 **Genes in GO geneset 'fatty acid metabolism'**. A tab separated text file that shows the identities and descriptions of the genes of the GO geneset 'fatty acid metabolism' present in the breast cancer microarray dataset. Click here for file

Additional file 6 **Genes in GO geneset 'receptor mediated endocytosis'**. A tab separated text file that shows the identities and descriptions of the genes of the GO geneset 'receptor mediated endocytosis' present in the lung cancer microarray dataset. Click here for file

Additional file 7 **Genes in GO geneset 'brain development'**. A tab separated text file that shows the identities and descriptions of the genes of the GO geneset 'brain development' present in the lung cancer microarray dataset. Click here for file

Additional file 8 **Genes in GO geneset 'apical plasma membrane'**. A tab separated text file that shows the identities and descriptions of the genes of the GO geneset 'apical plasma membrane' present in the lung cancer microarray dataset. Click here for file

Additional file 9 **Genes in KEGG geneset 'MAPK signaling'**. A tab separated text file that shows the identities and descriptions of the genes of the KEGG geneset 'MAPK signaling' present in the lung cancer microarray dataset. Click here for file

Additional file 10 **Stability analysis to assess the overall number of permutations using the GO genesets for breast cancer OS**. For each original GO geneset, an empirical p-value_2 _was calculated from different distributions based on different numbers of random genesets. The negative logarithms of the p-values_2 _to the base 10 from 100 k (i.e. 100,000) permutations are plotted against the negative logarithms of p-values_2 _to the base 10 from different permutations (A, B, C, D, and E). If the empirical p-values_2 _from all distributions were equal, an x = y line would be observed. As the number of random genesets used to model the distributions increase from 1 k to 50 k, the empirical p-values_2 _resemble those of the 100 k permutations (A, B, C, and D). Increasing the number of permutations from 100 k to 200 k does not considerably change the significance of the GO genesets (E). The empirical p-values_2 _obtained for the GO genesets from all the permutations show that empirical p-values_2 _from 200 k, 100 k, and 50 k distributions are all very consistent (F). Click here for file

Additional file 11 **Stability analysis to assess the overall number of permutations using the GO genesets for breast cancer RFS**. For each original GO geneset, an empirical p-value_2 _was calculated from different distributions based on different numbers of random genesets. The negative logarithms of the p-values_2 _to the base 10 from 100 k (i.e. 100,000) permutations are plotted against the negative logarithms of p-values_2 _to the base 10 from different permutations (A, B, C, D, and E). If the empirical p-values_2 _from all distributions were equal, an x = y line would be observed. As the number of random genesets used to model the distributions increase from 1 k to 50 k, the empirical p-values_2 _resemble those of the 100 k permutations (A, B, C, and D). Increasing the number of permutations from 100 k to 200 k does not considerably change the significance of the GO genesets (E). The empirical p-values_2 _obtained for the GO genesets from all the permutations show that empirical p-values_2 _from 200 k, 100 k, and 50 k distributions are all very consistent (F). Click here for file

Additional file 12 **Stability analysis to assess the overall number of permutations using the GO genesets for lung cancer OS**. For each original GO geneset, an empirical p-value_2 _was calculated from different distributions based on different numbers of random genesets. The negative logarithms of the p-values_2 _to the base 10 from 100 k (i.e. 100,000) permutations are plotted against the negative logarithms of p-values_2 _to the base 10 from different permutations (A, B, C, D, and E). If the empirical p-values_2 _from all distributions were equal, an x = y line would be observed. As the number of random genesets used to model the distributions increase from 1 k to 50 k, the empirical p-values_2 _resemble those of the 100 k permutations (A, B, C, and D). Increasing the number of permutations from 100 k to 200 k does not considerably change the significance of the GO genesets (E). The empirical p-values _2_obtained for the GO genesets from all the permutations show that empirical p-values_2 _from 200 k, 100 k, and 50 k distributions are all very consistent (F). Click here for file
