# Supplementary figures and images for: Modelling p-value distributions to improve theme-driven survival analysis of cancer transcriptome datasets
Source: BMC Bioinformatics. 2010 Jan 11;11:19. doi: 10.1186/1471-2105-11-19 (PMC2824674; doi:10.1186/1471-2105-11-19)

**(A) Biocarta-like genesets**

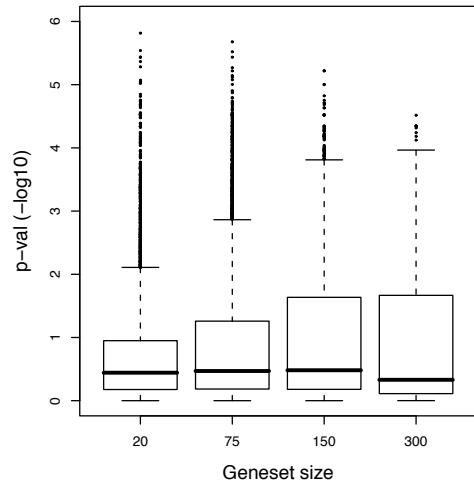

**(B) GO-like genesets**

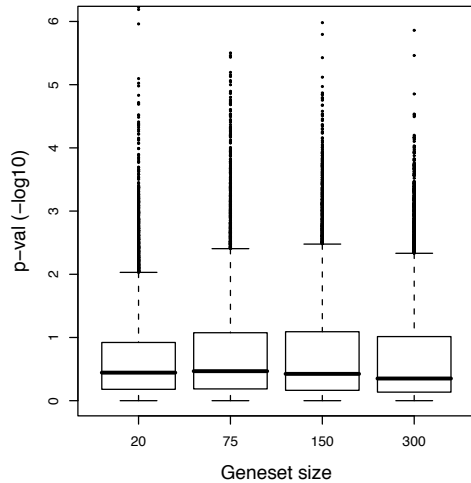

**(C) KEGG-like genesets**

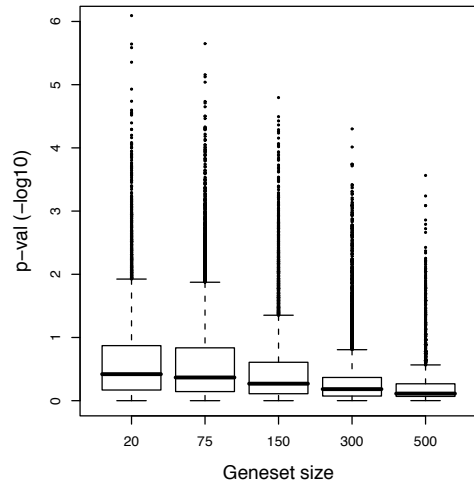

Supplement: Additional file 1 — Association between geneset size and significance for breast cancer RFS. For each type of geneset (i.e. Biocarta, GO, and KEGG), sets of random genes of sizes 20, 75, 150, 300, and 500 (the latter omitted for Biocarta because the pool of genes was too small) consisting of 10,000 genesets for each size were generated. For each geneset, hierarchical clustering was performed to segregate samples into two groups, a subsequent log-rank test was performed to assess a difference in prognosis between both groups, and the p-value1 was recorded. The negative base 10 logarithms of the p-values1 are plotted against geneset size. Biocarta-like genesets appear to be more significant around length = 150 (A); GO-like genesets do not show a clear correlation of significance to geneset size (B); KEGG-like genesets seem to be more significant as size becomes smaller (C). [file 1471-2105-11-19-S1.PDF]

**(A) Biocarta-like genesets**

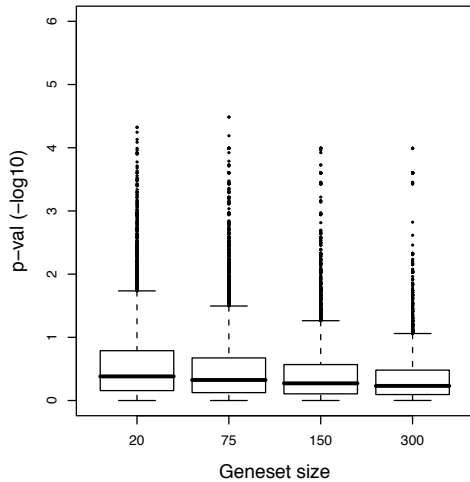

**(B) GO-like genesets**

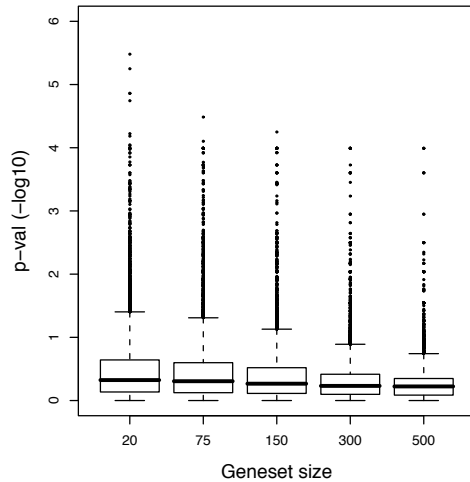

**(C) KEGG-like genesets**

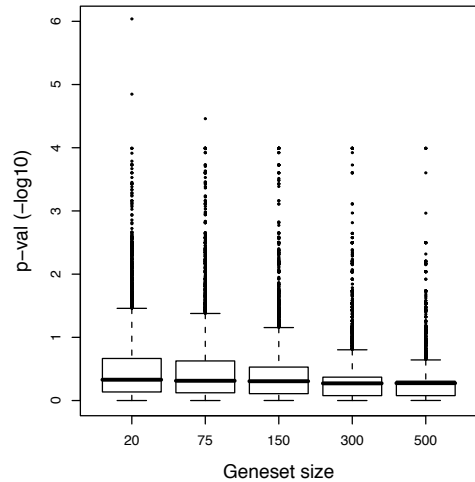

Supplement: Additional file 2 — Association between geneset size and significance for lung cancer OS. For each type of geneset (i.e. Biocarta, GO, and KEGG), sets of random genes of sizes 20, 75, 150, 300, and 500 (the latter omitted for Biocarta because the pool of genes was too small) consisting of 10,000 genesets for each size were generated. For each geneset, hierarchical clustering was performed to segregate samples into two groups, a subsequent log-rank test was performed to assess a difference in prognosis between both groups, and the p-value1 was recorded. The negative base 10 logarithms of the p-values1 are plotted against geneset size. Biocarta-like genesets seem to be more significant as size becomes smaller (A); GO-like genesets seem to be more significant as size becomes smaller (B); KEGG-like genesets seem to be more significant as size becomes smaller (C). [file 1471-2105-11-19-S2.PDF]

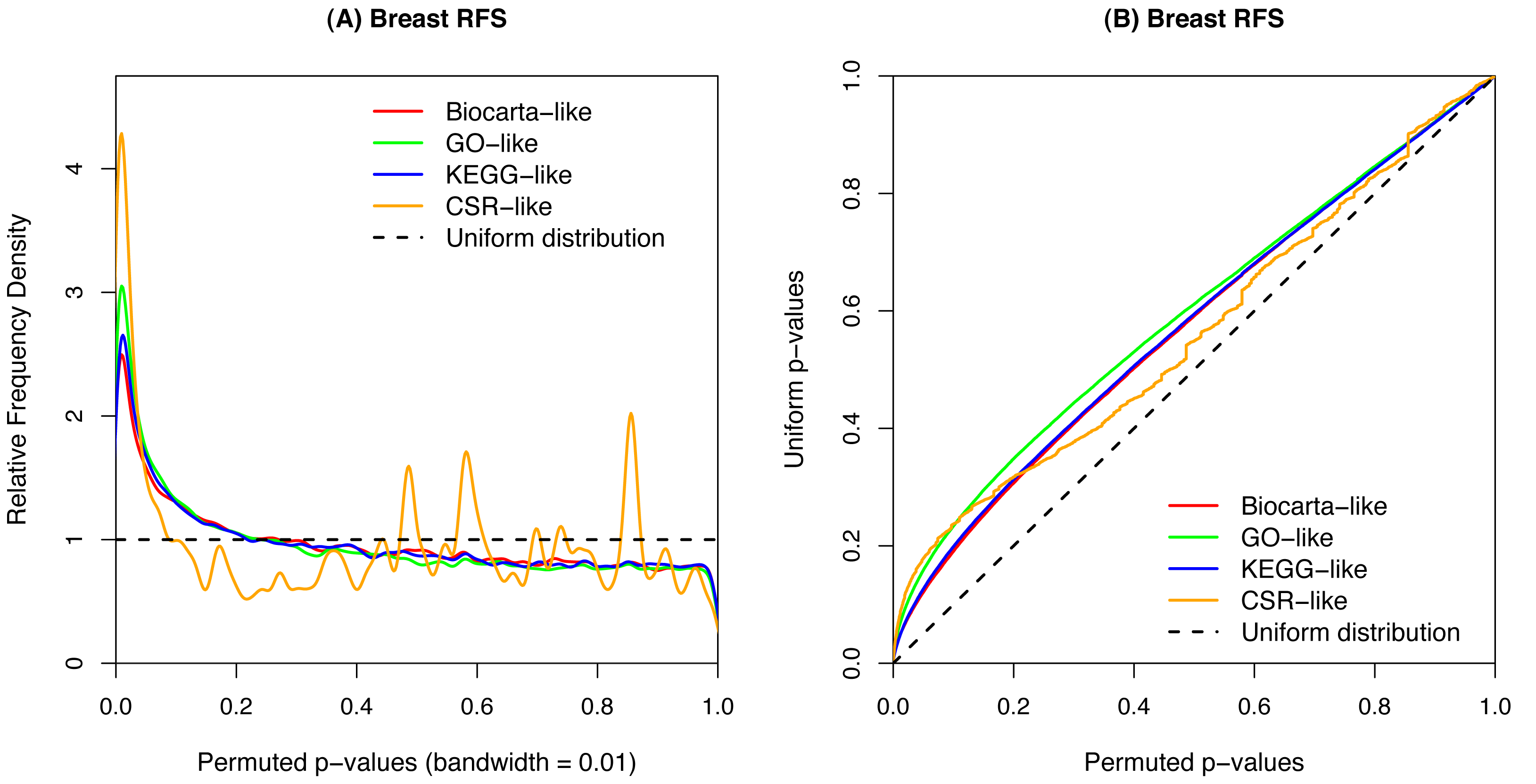

Supplement: Additional file 3 — Empirical p-value1distributions of random genesets for breast cancer RFS. Relative frequency density distributions are shown for breast cancer RFS (A). The relative frequency density estimate with a bandwidth equal to 0.01 is plotted for each empirical distribution (i.e. Biocarta-like, GO-like, KEGG-like, and CSR-like). A uniformly distributed empirical distribution would result in p-values1 at the same frequency across the entire range 0 < p < 1, (dashed line in (A)). An ordered plot of empirical p-value1 distributions versus the uniform distribution is shown for breast cancer RFS (B). The permuted p-values1 used to model each distribution (i.e. Biocarta-like, GO-like, KEGG-like, and CSR-like) are plotted against random p-values from a uniform distribution. An x = y line would be expected if the empirical distributions were uniformly distributed. [file 1471-2105-11-19-S3.TIFF]

**(A) Breast OS**

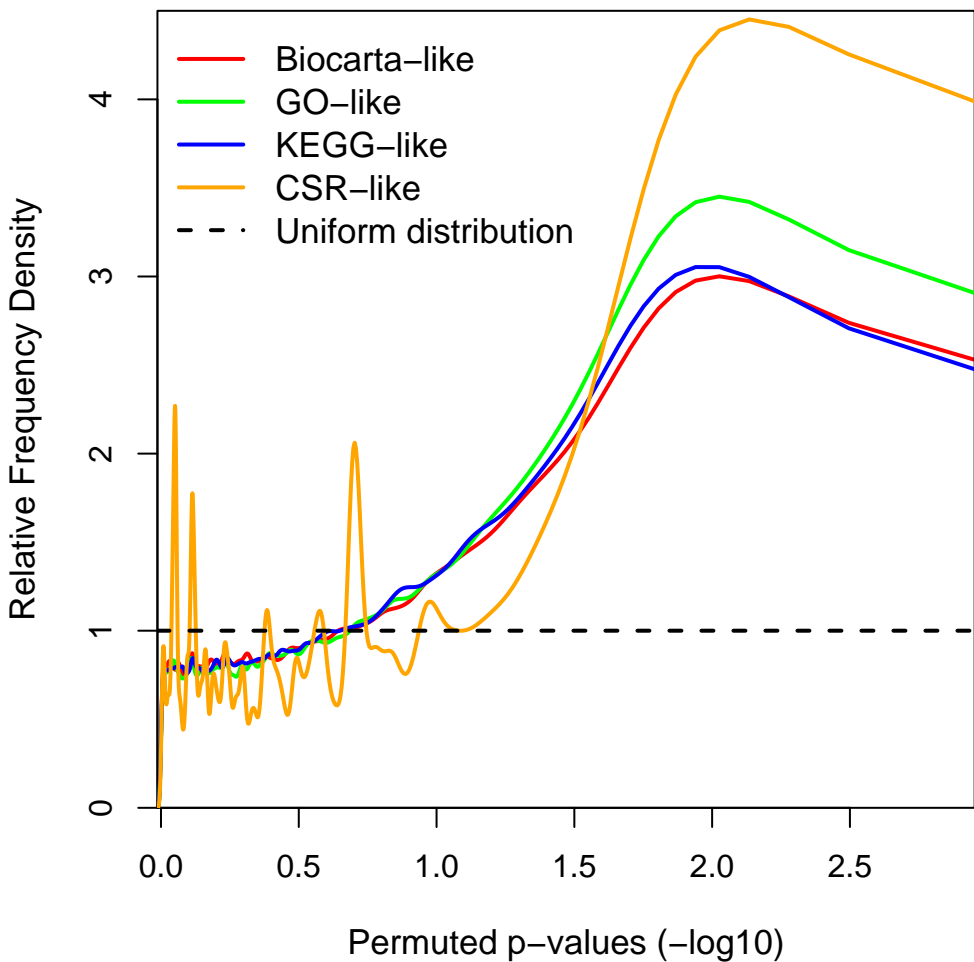

**(B) Lung OS**

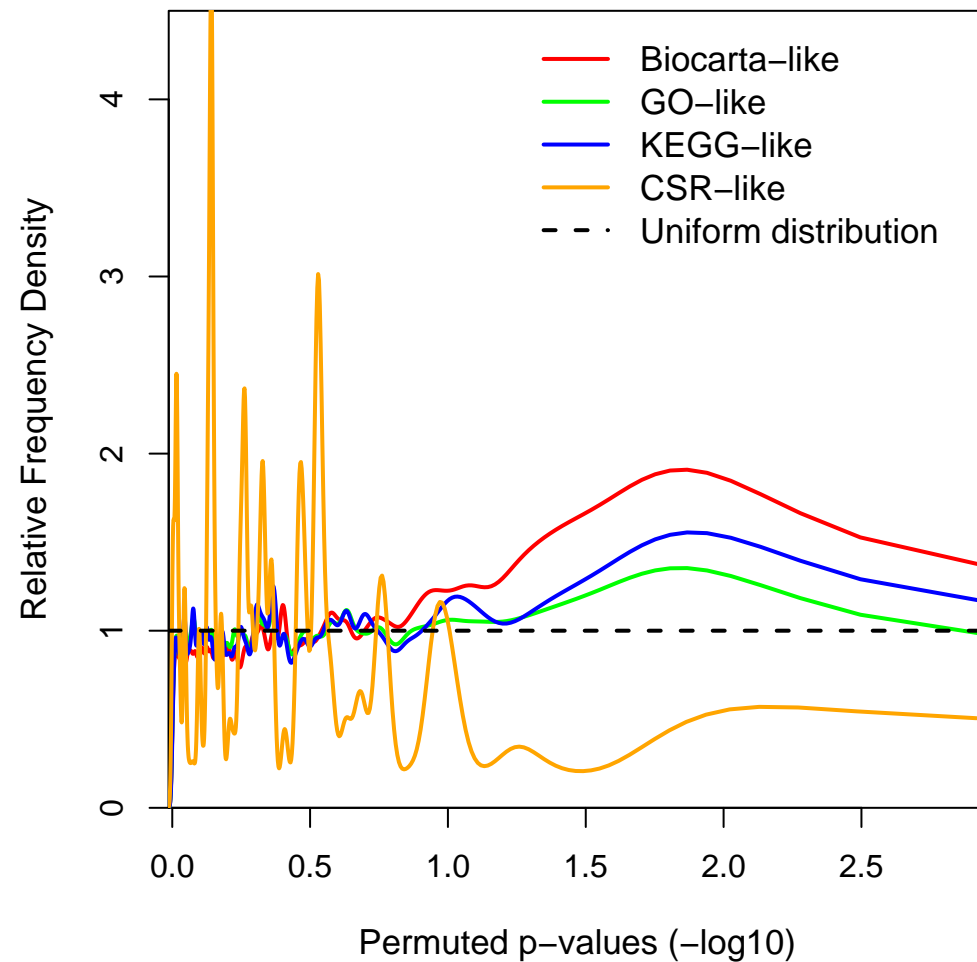

Supplement: Additional file 4 — Empirical p-value density distributions of random genesets for breast cancer OS and lung cancer OS (negative logarithmic scale). Relative frequency density distributions are shown for breast cancer OS (A) and for lung OS (B). For each survival estimate (i.e. breast cancer OS, and lung cancer OS), the relative frequency density estimate with a bandwidth equal to 0.01 is plotted for each empirical distribution (i.e. Biocarta-like, GO-like, KEGG-like, and CSR-like) on a negative logarithmic to the base 10 scale. A uniformly distributed empirical distribution would result in p-values1 at the same frequency across the entire range -log(0) > -log(p) > -log(1) (dashed line in (A) and (B)). [file 1471-2105-11-19-S4.PDF]

**(A)**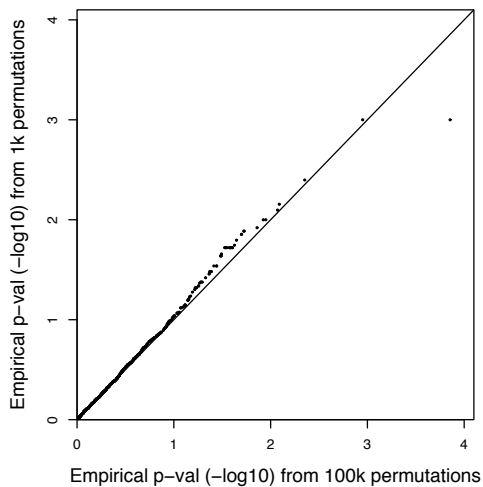**(B)**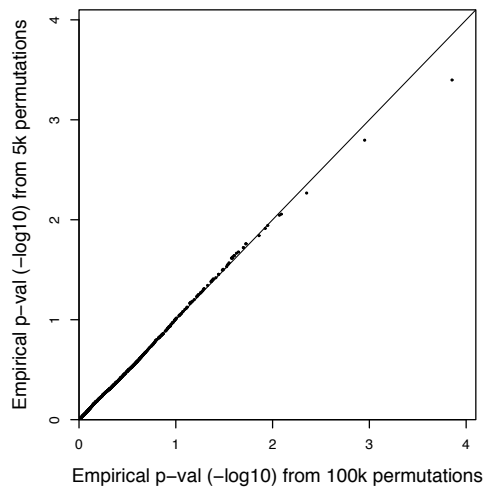**(C)**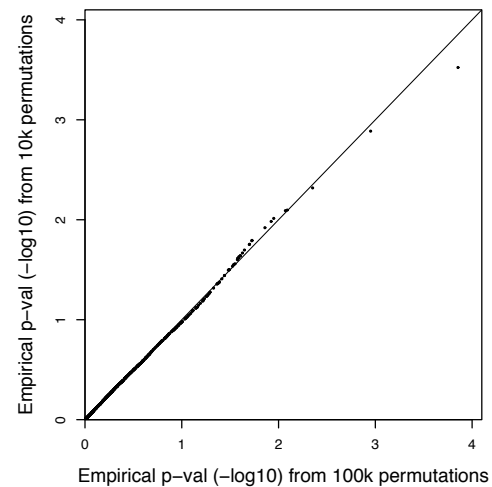**(D)**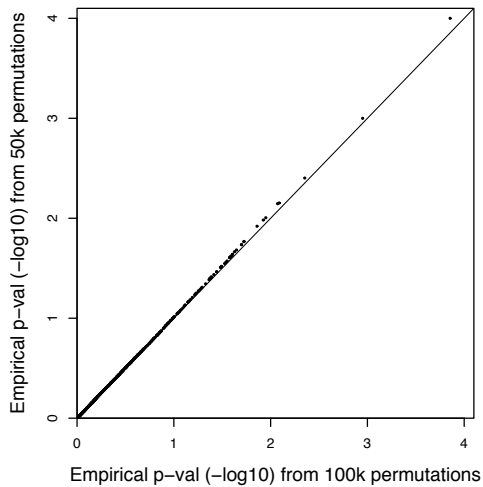**(E)**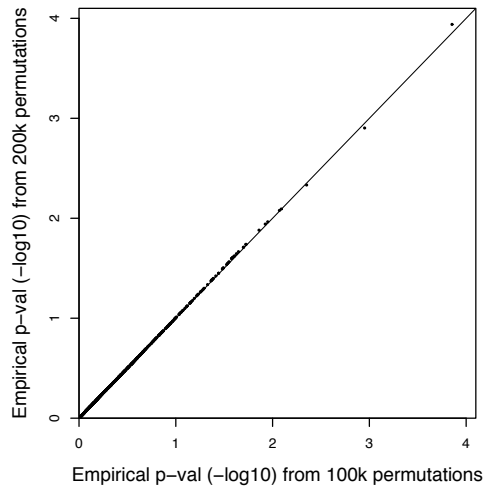**(F)**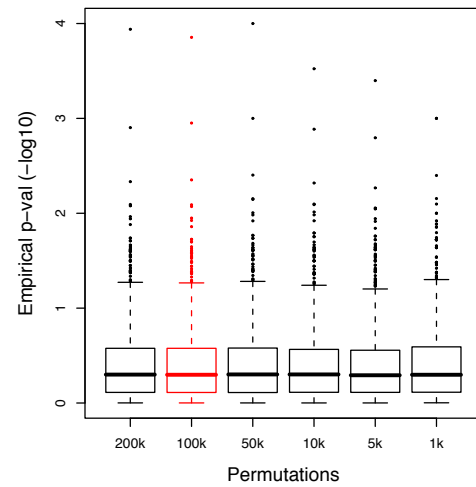

Supplement: Additional file 10 — Stability analysis to assess the overall number of permutations using the GO genesets for breast cancer OS. For each original GO geneset, an empirical p-value2 was calculated from different distributions based on different numbers of random genesets. The negative logarithms of the p-values2 to the base 10 from 100 k (i.e. 100,000) permutations are plotted against the negative logarithms of p-values2 to the base 10 from different permutations (A, B, C, D, and E). If the empirical p-values2 from all distributions were equal, an x = y line would be observed. As the number of random genesets used to model the distributions increase from 1 k to 50 k, the empirical p-values2 resemble those of the 100 k permutations (A, B, C, and D). Increasing the number of permutations from 100 k to 200 k does not considerably change the significance of the GO genesets (E). The empirical p-values2 obtained for the GO genesets from all the permutations show that empirical p-values2 from 200 k, 100 k, and 50 k distributions are all very consistent (F). [file 1471-2105-11-19-S10.PDF]

**(A)**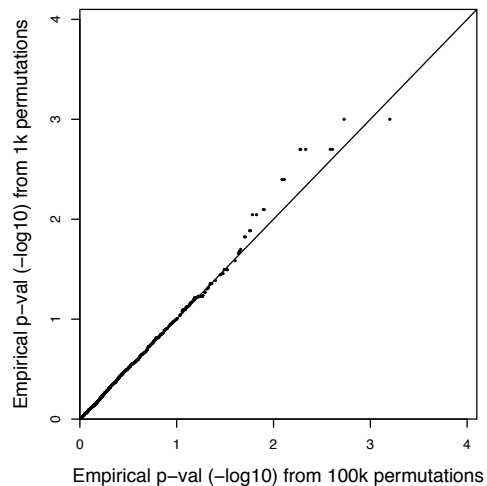**(B)**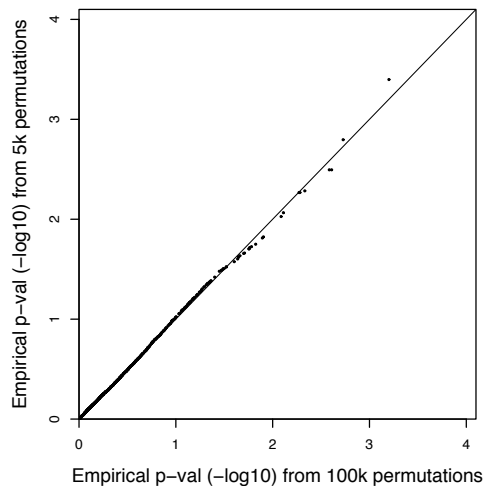**(C)**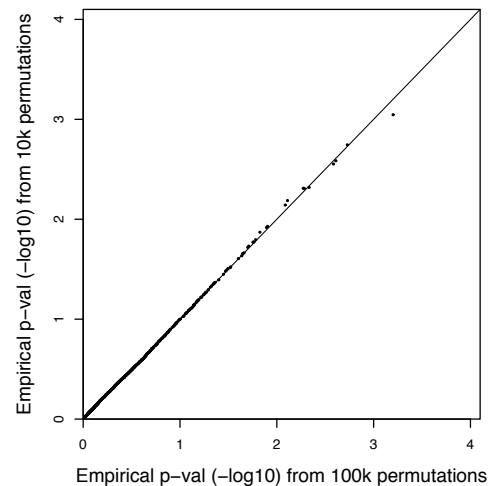**(D)**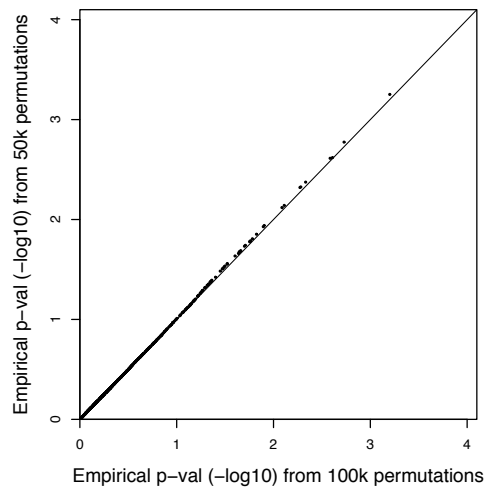**(E)**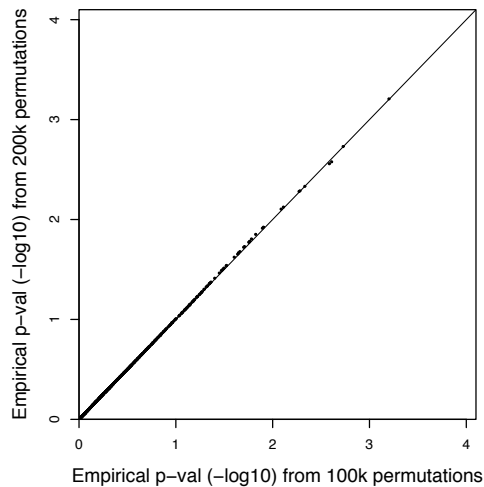**(F)**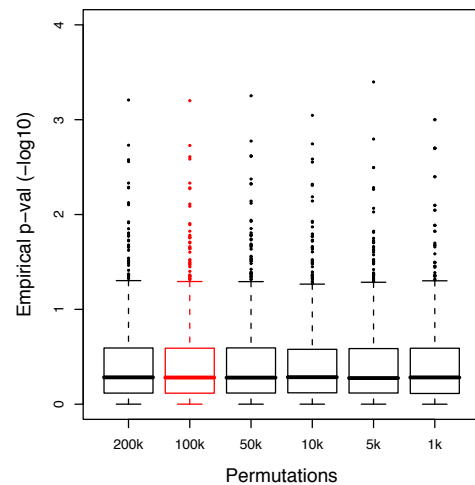

Supplement: Additional file 11 — Stability analysis to assess the overall number of permutations using the GO genesets for breast cancer RFS. For each original GO geneset, an empirical p-value2 was calculated from different distributions based on different numbers of random genesets. The negative logarithms of the p-values2 to the base 10 from 100 k (i.e. 100,000) permutations are plotted against the negative logarithms of p-values2 to the base 10 from different permutations (A, B, C, D, and E). If the empirical p-values2 from all distributions were equal, an x = y line would be observed. As the number of random genesets used to model the distributions increase from 1 k to 50 k, the empirical p-values2 resemble those of the 100 k permutations (A, B, C, and D). Increasing the number of permutations from 100 k to 200 k does not considerably change the significance of the GO genesets (E). The empirical p-values2 obtained for the GO genesets from all the permutations show that empirical p-values2 from 200 k, 100 k, and 50 k distributions are all very consistent (F). [file 1471-2105-11-19-S11.PDF]

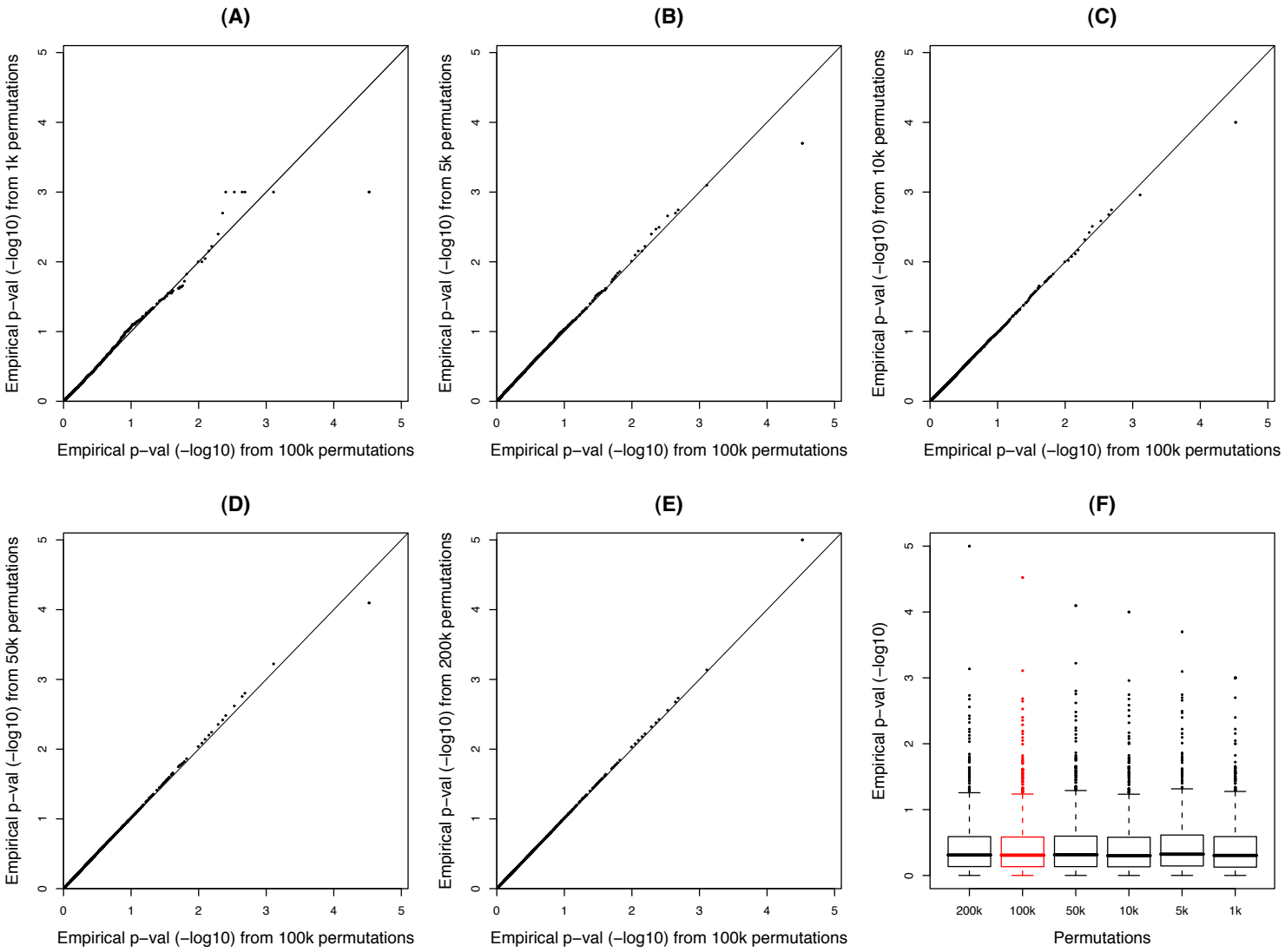

Supplement: Additional file 12 — Stability analysis to assess the overall number of permutations using the GO genesets for lung cancer OS. For each original GO geneset, an empirical p-value2 was calculated from different distributions based on different numbers of random genesets. The negative logarithms of the p-values2 to the base 10 from 100 k (i.e. 100,000) permutations are plotted against the negative logarithms of p-values2 to the base 10 from different permutations (A, B, C, D, and E). If the empirical p-values2 from all distributions were equal, an x = y line would be observed. As the number of random genesets used to model the distributions increase from 1 k to 50 k, the empirical p-values2 resemble those of the 100 k permutations (A, B, C, and D). Increasing the number of permutations from 100 k to 200 k does not considerably change the significance of the GO genesets (E). The empirical p-values 2obtained for the GO genesets from all the permutations show that empirical p-values2 from 200 k, 100 k, and 50 k distributions are all very consistent (F). [file 1471-2105-11-19-S12.PDF]
